# Practical Approaches to Nutritional Interventions for Malnutrition, Sarcopenia, and Frailty in Community-Dwelling Older Adults and Care Home Populations: A Narrative Review

**DOI:** 10.3390/nu18193189

**Published:** 2026-09-27

**Authors:** Haya Khalaf, Camilla Hopwood, Caryn Straker, Kirsty Donald, Nuno Lopes, Adrian Slee

**Affiliations:** 1 Division of Medicine, Faculty of Medical Sciences, University College London (UCL), London WC1E 6BT, UK; haya.khalaf.24@ucl.ac.uk; 2 AYMES International Ltd., Unit B, Sunnyhills Road, Leek ST13 5RJ, UK; chopwood@aymes.com (C.H.); kdonald@aymes.com (K.D.); 3 Nightingale Hammerson Care Home, London SW12 8NB, UK; dieteticpractitioner@nightingalehammerson.org; 4 KYN Hurlingham, 28 Daisy Lane, London SW6 3DD, UK; nuno.lopes@nihr.ac.uk

**Keywords:** malnutrition, sarcopenia, frailty, care homes, Food First approach, oral nutrition supplementation

## Abstract

Background: Average life expectancy has increased substantially on a global scale; this demographic shift presents considerable public health challenges, as increased longevity is accompanied by higher prevalence of chronic diseases, functional decline, and dependency. Malnutrition, sarcopenia, and frailty frequently coexist in older adults, particularly among care home residents. These interrelated conditions contribute to adverse clinical outcomes including falls, hospitalisation, and reduced quality of life. Despite their growing recognition, gaps remain in understanding optimal nutritional intervention strategies and their implementation in care home settings. Purpose and scope: This literature review examines the interplay between malnutrition, sarcopenia, and frailty in older adults, with particular focus on care home populations. The review evaluates evidence-based nutritional interventions, including the Food First approach, food fortification, and oral nutritional supplements (ONSs), and explores factors influencing intervention adherence and effectiveness. Main findings and conclusions: Evidence demonstrates that malnutrition, sarcopenia, and frailty share common pathophysiological mechanisms and overlapping clinical features such as unintended weight loss and age-related loss of muscle mass and strength yet represent distinct conditions requiring targeted assessment and intervention. While food fortification effectively increases energy and protein intake, when insufficient, ONS have been shown to significantly increase energy intake, BMI, as well as improve functional outcomes, reduce hospitalisation rates, and demonstrate cost effectiveness. However, adherence remains challenging and can be affected by barriers related to personal, contextual or product-related factors. Such nutritional strategies, particularly when combined with others such as physical activity/exercise as part of a multimodal approach, and focusing on providing an individualised, patient-centred care may help to achieve optimal outcomes in older adults. Further research is needed to establish standardised protocols for implementing combined nutritional and multimodal strategies in diverse care environments to optimise health outcomes for older adults.

## 1. Introduction

Global life expectancy has increased significantly, with most individuals now expected to live well beyond 60 years of age. By 2030, it is estimated that one in six people globally will be aged 60 years or over, increasing the population in this age group from 1 billion in 2020 to 1.4 billion [1]. This demographic shift is projected to accelerate further, with numbers effectively doubling within three decades and the older population is anticipated to reach 2.1 billion by the year 2050 [1].

While rising life expectancy across nearly all nations represents a significant achievement, this increased longevity comes with an increase in the prevalence of chronic disease, functional decline, and dependency, collectively leading to distinct public health challenges as recognised by both health professionals and policymakers [2]. For example, data reveal that in the United Kingdom (UK), one-fifth of individuals aged in their 50s suffer from multiple chronic health conditions (termed multimorbidity), with prevalence escalating with age. By age 80 years and older, approximately two-thirds (68%) of the population live with two or more chronic conditions [3].

Advanced age renders older adults particularly vulnerable to disease-related weight loss, sarcopenia, and frailty, all of which significantly compromise recovery and overall wellbeing [4]. Unintentional weight loss associated with disease and frailty, indicating macronutrient deficiency and/or heightened catabolism, commonly serves as an initial marker that triggers a catabolic cascade leading to increased morbidity and mortality [4]. This is particularly critical in care home settings, where residents represent one of the most nutritionally vulnerable populations. The frequent co-existence of malnutrition, frailty, and sarcopenia in this group significantly contributes to poor clinical outcomes and reduced quality of life (QoL) [5]. Consequently, the prevention and management of malnutrition in care home residents remains a key public health and clinical priority [5]. As per data and analysis from Census 2021, in England and Wales, 278,946 individuals aged 65 years and over resided in care homes, representing 82.1% of the total care home population [6]. Additionally, data from the Department of Health and Social Care (2025) indicated that 86.3% of care home beds in England were occupied during the week ending 14 November 2025 [7]. Overall, these findings underscore the essential role of quality health and care service provision in enabling older adults to maintain wellbeing.

This literature review aims to synthesise current evidence on the interconnection between malnutrition, sarcopenia, and frailty in older adults, with particular focus on care home populations. The review critically examines Food First approaches and oral nutritional supplements (ONSs) as key nutritional interventions, evaluating their efficacy through critique of existing evidence, identifying current themes, implementation barriers, and gaps in knowledge to inform future care and research in this vulnerable population. A literature review was performed using key and current literature providing an overview of the topic. Specifically for the nutritional intervention sections, a comprehensive literature search was conducted on PubMed and Google Scholar databases, and abstracts of relevant articles were examined. Furthermore, a search was performed including combining terms such as “malnutrition” and “protein” with “nursing homes,” “care homes,” or “geriatric nutrition”. Articles on interventions published between 2013 and 2026 that met the aim of the present review were included.

## 2. Defining the Triad of Malnutrition, Frailty, and Sarcopenia

### 2.1. Malnutrition and Ageing

The definition and assessment of malnutrition has evolved considerably over time, with multiple diagnostic approaches and criteria proposed across the literature [8]. Although numerous attempts have been made, consensus on a single, objective definition has remained elusive. To address this definitional gap, an international consensus process was initiated in 2016, resulting in the Global Leadership Initiative on Malnutrition (GLIM) criteria [4,9]. According to the GLIM, malnutrition arises through two principal pathways: undernutrition, attributable to compromised nutrient intake or absorption, and disease-related malnutrition, which encompasses varying degrees of acute or chronic inflammation in conjunction with reduced dietary intake or impaired nutrient assimilation leading to altered body composition and diminished biological function [9]. Inflammation contributes to these changes through increased resting energy expenditure and accelerated muscle catabolism, ultimately manifesting as measurable reductions in fat-free mass (FFM), muscle mass index, or body cell mass, which are viewed as “critical dynamic protein reserves”. Together, these alterations are associated with adverse functional and clinical outcomes [9].

Physiological changes associated with aging, such as decreased sensory perception of smell and taste, diminished appetite (“anorexia of ageing”), and shifts in dietary habits and food choices, often result in suboptimal food intake [10]. This is further compounded by acute illness, medication use, poor oral health (including tooth loss and ill-fitting dentures), visual impairment, and gastrointestinal disorders, all of which can exacerbate anorexia [10]. It is estimated that anorexia of ageing prevalence ranges from 22% among community-dwelling older individuals to over 30% in care home populations and as high as 42% among those requiring acute hospitalisation [11]. Furthermore, social and environmental factors such as living or eating alone, caregiving responsibilities, limited access to food, reduced capacity or motivation to prepare meals, illness, and financial constraints may significantly impact nutritional status in this population [10].

In the UK, it is estimated that one in ten individuals aged 65 and over are affected by malnutrition [12]. This is further supported by findings from the British Association for Parenteral and Enteral Nutrition (BAPEN) Malnutrition and Nutritional Care Survey of Adults (2022), which reported that nearly half of all assessed individuals were identified as being at risk of disease-related malnutrition. When stratified by care setting, the highest prevalence of malnutrition risk was observed among older adults living in their own homes (56%) and care home residents (55%), followed by those in hospital settings (44%) [13].

### 2.2. Sarcopenia

Sarcopenia, referred to as a “muscle disease” or muscle failure, is defined by the European Working Group on Sarcopenia in Older People (EWGSOP) as a progressive generalised skeletal muscle disorder associated with ageing and an elevated risk of adverse outcomes, including falls, fractures, physical disability, and increased mortality [14,15]. Age-related skeletal muscle atrophy is considered to be an inevitable aspect of human aging [16]. This progressive decline begins at approximately 50 years of age and continues throughout the lifespan, resulting in approximately 50% fibre loss in limb muscles by 80 years of age, with an associated loss of motorneurone activity and reduction in type II, fast-twitch fibre cross-sectional area, and hence protein content [16]. However, the degree of atrophy affecting the remaining fibres is substantially determined by an individual’s typical physical activity levels [16]. It is worth noting that age-related muscle mass loss (sarcopenia) and strength loss (dynapenia) should be defined as distinct processes [17]. This distinction is supported by evidence showing that strength deterioration occurs much faster than muscle mass decline in ageing, with changes in quadricep muscle area accounting for only 6–8% of the variation in knee extensor strength changes [17]. Additional data confirm that muscle mass loss accounts for less than 10% of weakness associated with disuse, and gaining or preserving muscle mass does not prevent age-related strength decline [17]. These findings indicate that muscle weakness in older adults is more closely linked to impaired neural activation and/or diminished intrinsic force generating capacity and muscle quality, than to lean mass reduction [17].

Furthermore, the presence of chronic diseases significantly accelerates muscle depletion, with cardiovascular disease and chronic obstructive pulmonary disease (COPD) being notable contributors [18]. The chronic inflammation and metabolic abnormalities characteristic of these conditions enhance muscle catabolism and worsen sarcopenia progression. Additionally, sarcopenia prevalence is markedly higher among individuals with liver disease and stroke compared to their healthy counterparts [18]. However, the two principal factors associated with sarcopenia are malnutrition and physical inactivity [19]. Therefore, combined interventions incorporating appropriate exercise programmes and nutritional support represent essential strategies for optimising outcomes in sarcopenic geriatric patients [19].

Within the updated EWGSOP2 framework, low muscle strength alone is considered indicative of sarcopenia, while a confirmed diagnosis requires the concurrent presence of both low muscle strength and reduced muscle quality or quantity. Severe sarcopenia is identified when poor physical performance is also present [15].

The literature reports on sarcopenia prevalence show considerable variation, likely stemming from differences in the population studied (community-dwelling individuals, nursing home residents, or hospitalised patients), the diagnostic criteria applied, and the varied assessment methods used to measure muscle mass, strength, and physical performance [20]. A systematic review and meta-analysis including 16 studies with a total of 3585 participants from 129 nursing homes reported a sarcopenia prevalence of 41% among nursing home residents [21]. This is approximately four times greater than the 10% observed in another 2017 systematic review and meta-analysis conducted focusing on community-dwelling populations [22]. Additionally, a 2020 systematic review and meta-analysis including 41 studies with a total of 34,955 participants reported that nursing home residents exhibited the highest sarcopenia incidence (38%), compared to hospitalised individuals (23%) and community-dwelling older adults (10%) [20].

### 2.3. Frailty

Frailty, considered one of the most challenging aspects of ageing and frequently coexisting with malnutrition and sarcopenia, is defined as a clinical syndrome characterised by diminished physiological reserve and increased vulnerability to adverse health outcomes [23]. This could be a result of age-related hormonal changes, such as decreased production of anabolic hormones such as testosterone, and growth hormone/insulin-like growth factor-1 and increased catabolic hormone activity, particularly cortisol [24]. Such hormonal imbalances suppress muscle growth and increase muscle breakdown, which may compromise the body’s ability to recover from stressors and increase susceptibility to frailty [24]. The frailty phenotype is identified by the presence of three or more of the following five criteria: unintentional weight loss (10 lbs or more in the past year), self-reported exhaustion, weakness (measured by grip strength), slow walking speed, and low physical activity levels [23].

Within the ageing population, the prevalence of frailty is remarkably high, affecting approximately 10% of individuals aged 65 and over, with rates increasing alongside age and showing a higher frequency among women than men [25]. In addition, it is also linked to socioeconomic disadvantage, including lower education and income levels, as well as poorer health outcomes, greater chronic disease burden, and higher disability rates [23,25]. Frailty is progressively being understood as a screening framework to identify those at elevated disability risk, with potential for improvement through both targeted and general interventions such as nutrition and exercise [26,27].

The clinical manifestations of frailty, especially within the physical domains, exhibit substantial overlap with those of sarcopenia [28]. Sarcopenia, regarded as muscle organ failure or insufficiency, commonly serves as an underlying cause of physical frailty. The inclusion of diminished physical performance within sarcopenia indicates its essential role as a component of physical frailty [26]. The convergence of these two conditions was inevitable given that both address common subclinical and clinical features of ageing [28]. Additionally, malnutrition is recognised as a critical contributing factor in the development of both conditions [26]. Common pathophysiological pathways link malnutrition and physical frailty, and their respective screening tools incorporate overlapping criteria such as weight loss and impaired physical function. Malnutrition holds a central position within the physical frailty phenotype conceptualised by Fried et al., with weight loss regarded as a modifiable contributor to frailty [29]. In a 2018 meta-analysis investigating the association between malnutrition and physical frailty among community-dwelling older adults, it was found that two-thirds of malnourished individuals were frail, while 10% of frail individuals were malnourished, suggesting that the two conditions are related but not interchangeable geriatric syndromes [29]. Figure 1 below depicts the overlap between conditions with shared clinical factors.

**Figure 1:**
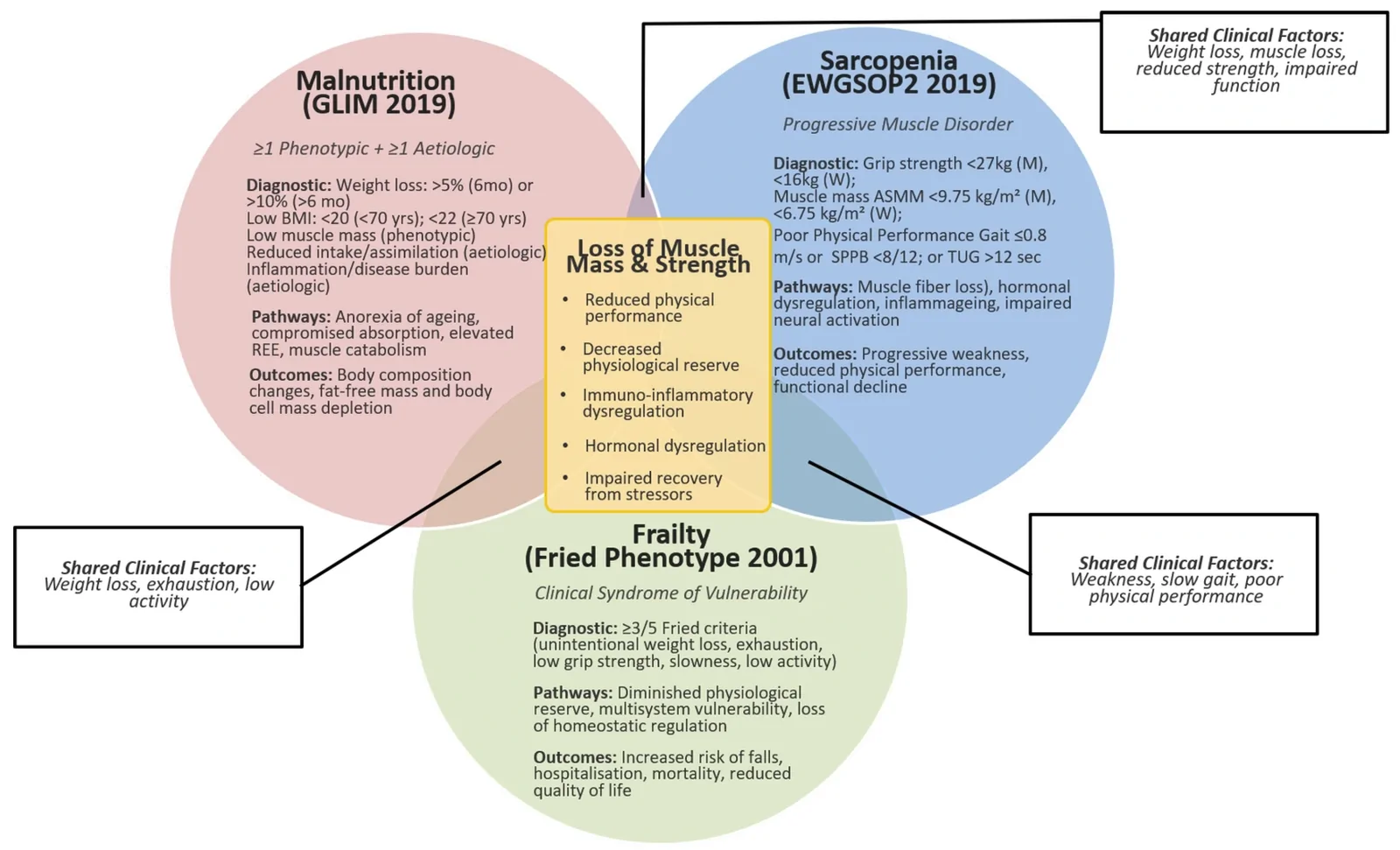
The overlapping triad of malnutrition, sarcopenia, and frailty. The three overlapping circles represent malnutrition, sarcopenia, and frailty, three interconnected but distinct geriatric syndromes prevalent in older adults, particularly care home residents. The overlap between malnutrition and sarcopenia is driven by shared mechanisms including inadequate protein intake, anorexia of ageing, weight loss, and disease-related inflammation, which collectively accelerate muscle protein catabolism and reduce fat-free mass. The overlap between sarcopenia and frailty reflects their strong biological relationship, wherein sarcopenia serves as a fundamental biological substrate and primary driver of physical frailty. The overlap between malnutrition and frailty reflects their epidemiological association; however, malnutrition and frailty represent distinct syndromes, with malnutrition primarily reflecting inadequate nutrient intake and frailty encompassing broader physiological vulnerability along with key aspects such as associations with reduced physical activity and disease risk, loss of muscle mass and function becomes inevitable, leading to reduced observations in gait speed, endurance/exercise capacity, and muscle weakness. Yet, frailty is also associated with the anorexia of ageing and reduced food intake and hence reduced protein and energy intake. Although other crucial overlapping factors such as chronic inflammation, hormonal dysregulation, and reduced physical activity may also contribute to the development of all three conditions, this review emphasises loss of muscle mass and strength as the critical link warranting an emphasis on evidence-based interventions such as nutrition in older adults, particularly care home residents. Abbreviation: GLIM, Global Leadership Initiative on Malnutrition; EWGSOP2, European Working Group on Sarcopenia in Older People 2; ASMM, Appendicular Skeletal Muscle Mass; REE, Resting Energy Expenditure.

### 2.4. Nutritional Screening

Given these adverse consequences, routine assessment and monitoring of malnutrition using validated screening tools early is essential in older populations [30]. The conceptual confusion between nutritional screening and assessment has historically hindered standardised malnutrition diagnosis. The GLIM criteria address this by introducing a standardised, internationally recognised framework for diagnosing malnutrition using a two-step approach [9]. The first step involves risk screening using validated tools. Individuals identified as at risk then undergo diagnostic assessment based on the presence of at least one phenotypic and one aetiologic criterion. Phenotypic criteria include unintentional weight loss, low body mass index (BMI), and reduced skeletal muscle mass (the latter reflecting the growing recognition of sarcopenia as a key component of malnutrition). Aetiologic criteria include reduced food intake and the presence of disease burden or inflammation [9,31].

An effective screening procedure should be standardised for widespread implementation, characterised by efficiency, simplicity, and practicality, and rely on parameters that are readily available and accessible [32]. Among the screening tools with established validity for use in older populations are the Nutritional Risk Screening 2002 (NRS-2002), Malnutrition Universal Screening Tool (MUST), Mini Nutritional Assessment (MNA), Mini Nutritional Assessment–Short Form (MNA-SF), Malnutrition Screening Tool (MST), and Subjective Global Assessment (SGA) [33]. In the UK, the MUST tool is the standard, validated tool developed by BAPEN (British Association for Parenteral and Enteral Nutrition) and endorsed by National Institute for Health and Care Excellence (NICE) for use across all care settings [34,35]. However, the MNA is the most widely utilised and validated screening tool for nutritional assessment in older adults and is applicable across all geriatric care settings [36]. To further minimise administration time, Rubenstein et al. developed the six-item MNA-SF by selecting a subset of questions from the full MNA that demonstrated high sensitivity, specificity, and strong correlation with the complete assessment tool [37]. This abbreviated version is particularly useful in older care home populations where time efficiency and ease of administration are essential [38].

A comparison of MUST and MNA-SF demonstrates that the two instruments evaluate malnutrition risk using different approaches and frameworks [5]. MUST is limited to objective physical criteria such as BMI, unintentional weight loss, and acute disease whereas the MNA-SF adopts a broader, multidimensional perspective. In addition to BMI, it incorporates mobility, neuropsychological conditions such as depression and dementia, and the presence of psychological stress or acute illness [5]. These domains overlap substantially with components of frailty, and previous research has shown that the MNA-SF can predict both pre-frailty and frailty [39,40]. In addition, MNA-SF applies a higher BMI cut-off consistent with the Global Leadership Initiative on Malnutrition recommendations (<22 kg/m^2^ for adults > 70 years in Europe). Collectively, these features enable the MNA-SF to identify broader determinants of malnutrition risk that are not captured by MUST [5].

Furthermore, a key phenotypic criterion in the GLIM framework for malnutrition diagnosis is reduced muscle mass [9]. Various techniques exist for assessing muscle quantity or mass, with results adjustable for height or BMI [15]. Although dual-energy X-ray absorptiometry (DEXA) has long been considered the gold standard for body composition assessment, computed tomography (CT) and magnetic resonance imaging (MRI) have also proven accurate and reliable [41]. These methods, however, face practical limitations including high costs, radiation exposure, and restricted accessibility. In contrast, bioelectrical impedance analysis (BIA) offers a more feasible alternative, providing a non-invasive, cost-effective approach with high reproducibility and ease of implementation [15,41]. However, significant muscle mass loss, regardless of cause, is typically accompanied by impaired muscle function [9]. When direct muscle mass assessment is not feasible for direct sarcopenia diagnosis, muscle strength measurements, such as handgrip strength using a dynamometer, may offer appropriate proxy measures [9,31].

### 2.5. Nutritional Requirements: The Energy and Protein Requirement Gap

Meeting energy and protein requirements in older adults living in long-term care settings remains challenging. Resting energy expenditure (REE) typically declines with advancing age, primarily as a consequence of reduced fat-free body mass [30]. In older adults with illness, energy needs may decrease secondary to reduced physical activity, while concurrently increasing due to disease processes including inflammation, fever, and medication effects [30]. A general guideline of approximately 30 kcal/kg body weight (BW) is recommended as an initial estimate for energy requirements in older individuals, although individualisation and tailoring based on relevant clinical and physiological factors is essential [30].

The PROT-AGE Study Group, a collaborative initiative of the European Union Geriatric Medicine Society (EUGMS) and associated organisations, developed evidence-based recommendations for protein consumption in older adults, taking into account the numerous physiological changes that occur with ageing. The guidelines recommend a daily protein intake of 1.0–1.2 g/kg BW to maintain or restore age-related losses in lean body mass and physical function. Higher intake of 1.2–1.5 g/kg BW per day is advised for older individuals with acute or chronic conditions, while those experiencing severe illness, injury, or significant malnutrition may require intake as high as 2.0 g/kg BW per day [42]. Likewise, The European Society for Clinical Nutrition and Metabolism (ESPEN) guidelines recommend protein intake of at least 1.0–1.2 g/kg/day for healthy older adults, increasing to 1.2–1.5 g/kg/day in those who are malnourished or at risk, and higher intake of up to 2.0 g/kg BW a day in case of severe illness, injury, or malnutrition [30].

There is a clear and established association between inadequate protein intake and frailty in older adults, emphasising the necessity of increasing protein consumption for older adults in care home settings who are at heightened malnutrition risk [43]. Evidence from a 2023 Dutch nursing home study revealed that more than 50% of participants failed to meet the minimum protein recommendation of 0.83 g/kg BW per day, with only 15.3% consuming the suggested 1 g/kg BW per day, well below the 1.2–1.5 g/kg BW per day advised for frail older adults. These findings indicate that protein intake was markedly inadequate for most residents, regardless of which recommended threshold was applied [44]. Another multidepartment cross-sectional study conducted in south-central France revealed that the energy and protein intake of 83.8% of nursing home residents aged 75 years old and above was lower than the minimum requirement of 30 kcal/kg/day and 1.2 g/kg/day, respectively [45].

A further point to consider is the discussion over whether there are any additional benefits (e.g., net protein anabolism) of consuming large amounts of protein in one to two meals over spreading roughly equal amounts of protein throughout the day over several meals and snacks. A 2018 RCT conducted on older adults over an 8-week period comparing the two protein feeding patterns on older adults concluded that no significant differences were observed in lean body mass, muscle strength, or functional outcomes nor were there differences in whole-body net protein balance or muscle protein synthesis between the dietary approaches [46]. However, consuming large protein quantities within one or two meals may be impractical for long-term adherence, particularly among older populations when considering factors such as cost, physical limitations (e.g., dysphagia), and diminished appetite [46], whereas, encouraging higher protein intake at each meal may help older adults meet recommendations with greater ease [47]. Since the total quantity of protein consumed is more critical than the distribution pattern for anabolic response, it is reasonable to advise older adults to distribute their protein intake according to personal preference and practical feasibility [46].

Alternatively, when protein quantity cannot be increased in older adults due to post-meal satiety limits, focusing on protein quality may offer a more attainable approach. Protein quality is largely determined by essential amino acid content, particularly leucine, which holds unique anabolic and anti-catabolic properties [48]. Thus, enhancing nutritional intake through high-quality protein sources (such as beef, skimmed milk, eggs, and poultry), food fortification, and/or ONSs could potentially reduce disuse-related disruptions in muscle protein metabolism that contribute to muscle atrophy [49]. Moreover, the ageing process is associated with progressive immune dysfunction, characterised by chronic inflammation and diminished capacity to recognise new antigens, which increases susceptibility to infection and disease in older populations [50]. This decline directly impacts protein requirements, as amino acids are essential for immune system function and pathogen response. Key amino acids including glutamine, leucine, isoleucine, and valine are necessary for metabolic adaptations that enable immune cell proliferation and pathogen response as well as critical for hepatic acute phase protein and endogenous cellular glutathione production [50]. Additionally, enhanced protein processing methods (e.g., mincing meat) may help to improve amino acid availability, digestibility, and absorption from whole food protein sources, which may be particularly beneficial for older adults [49].

## 3. Nutrition Interventions

### 3.1. The “Food First” Approach: Fortification and Practical Approaches

Commonly, the standard approach to addressing malnutrition begins with a “Food First policy”. For example, in the UK, it is the first-line nutritional care strategy endorsed by both BAPEN and the British Dietetic Association (BDA) for managing disease-related malnutrition [51]. As outlined in the ESPEN practical guidelines on clinical nutrition and hydration in geriatrics (2022), this strategy focuses on fortification such as the addition of protein and energy-dense ingredients to standard portions without increasing volume as well as the modification of eating patterns [30]. Practical techniques include food-based fortification which involves the addition of fortifying nutrient-rich ingredients, to commonly eaten foods to intentionally increase dietary nutrient density without markedly expanding portion sizes [52]. This strategy is considered to be an effective intervention for older individuals with reduced appetite. Fortifying ingredients can be categorised as either whole food products (including semolina, oils, butter, cream, pureed nuts, and eggs) or macronutrient preparations (including whey protein isolate, milk protein concentrate, caseinates, and maltodextrin) [52]. Other techniques include the small frequent meals approach, advocating for 5–6 small meals or snacks per day to maximise intake in residents with small appetites [30]. This includes providing additional snacks possibly in the form of finger foods which help facilitate dietary intake particularly in individuals with dexterity issues such as difficulty using cutlery or prolonged sitting during mealtimes [30]. Moreover, for individuals with swallowing difficulties or impaired chewing, foods with modified consistency and enhanced nutritional content could be provided as a therapeutic approach [30]. Food fortification may also offer a flexible approach that may better align with the dietary preferences and established eating patterns of older adults. Through this method, older individuals or their carers can integrate fortifying ingredients into their accustomed meals during daily food preparation. This adaptability offers a considerable benefit in this population, as they may typically display some level of resistance to dietary changes [52]. Table 1 below summarises the food based fortifying ingredients, their clinical evidence and practical barriers.

Evidence suggests that the Food First strategy may help increase energy and protein intake in older adults, but it may not be adequate for all residents, particularly those with advanced frailty, or inflammatory disease conditions, and physiological barriers which may limit the effectiveness of dietary modification alone. A 2017 pilot study conducted in the Netherlands investigated protein intake enhancement in institutionalised older adults using a variety of protein-fortified food and beverage products, including commonly consumed items such as bread, soup, fruit juices, and instant mashed potatoes. The intervention aimed to increase participant protein intake to 1.2 g/kg BW per day. Over the 10-day intervention period, protein intake increased by an average of 11.8 grams per day. By the end of the study, all older adults achieved a minimum protein intake of 0.8 g/kg/day, with more participants meeting the recommended intake of 1.2 g/kg/day compared to baseline (nine versus four participants) [53]. Likewise, a systematic review conducted in 2017 examined 10 studies and found that food fortification was linked to higher energy intake in eight studies, greater protein intake in five studies, and weight gain in three studies [54]. Although such studies confirm that incorporating protein-fortified foods effectively increases protein consumption in older adults, additional research is required to assess its effects on muscle function and overall nutritional status. However, assessing muscle health, functional status, and body composition can be challenging in care home residents due to frailty, limited mobility and practical resources (e.g., accurate assessment equipment and trained operators). Thus, using adaptable outcome measures suited to institutional settings is critical. For example, a 2017 Finnish study involving 224 care home residents who were malnourished or at risk of malnutrition evaluated the effectiveness of a six-month nutritional intervention. The intervention comprised individualised dietary counselling designed to enhance energy-dense food consumption and increase meal frequency through the addition of nutrient-rich snacks and supplementary meals. Nutritional status was assessed using the MNA screening tool, BMI, and plasma albumin levels. The six-month nutritional intervention resulted in significantly improved nutritional outcomes in the treatment group, with MNA scores rising 2.3 points and serum albumin increasing 1.6 g/L, whereas the control group experienced marginal decreases in these markers, with MNA scores declining 0.2 points and albumin levels decreasing 0.1 g/L [55]. However, it is noteworthy that in this study, ONSs were provided to participants with inadequate energy or protein intake who were unable to achieve nutritional targets through dietary modification alone. Furthermore, a systematic review of 44 studies including the Pölönen, S., et al. (2017) study was conducted to review the impact of multiple diet enrichment strategies on nutritional outcomes [52]. When considering the impact of food-based fortification alone (27 studies out of 44), it was found that protein-fortified foods significantly improved protein intake in the majority of studies (16 out of 18), and energy-fortified foods increased energy intake in most cases (9 out of 13). However, the relative impact on nutritional status was less consistent, with limited studies showing significant improvements in MNA scores (1 out of 5), BW or BMI (4 out of 13), and muscle mass (1 out of 3) [52]. Although evidence suggests that food fortification reliably increases energy and protein intake, its effect on muscle mass, physical function, and frailty remains limited. For example, weight gain alone may be an insufficient indicator of nutritional benefit, particularly when its composition is unknown and may predominantly reflect increased fat mass, which does not necessarily translate into improved muscle strength or functional benefit. Furthermore, there is a need for clearer and more consistent definitions and reporting of food-based fortification, distinguishing it from other enrichment approaches such as ONS, while also evaluating preparation methods and acceptability. Given that the majority of the current evidence is derived from community settings, the effectiveness and feasibility of these approaches among care home populations who have greater nutritional vulnerability remains uncertain. Nevertheless, it is also worth noting that although enrichment strategies are typically presumed to be more economical than industrial nutritional preparations, more complex fortification strategies may result in increased costs when accounting for staff time and labour [56].

**Table 1:** Evaluating food-based fortifying ingredients for older adults: Clinical evidence and practical barriers.

Category	Examples of Fortifying Ingredients	Key Advantages	Quality of Evidence	Practical Implementation Barriers (Community vs. Residential Care)
Whole food fats	Butter, cream, oils (e.g., olive, soybean)	High energy density, improves palatability and "mouthfeel" of food.	High *(Mentioned in the ESPEN 2022 guidelines [30] and Geny et al., 2023 systematic review of literature [52])*	Community: Potential risk of exacerbating dyslipidaemia or cardiovascular concerns without regular clinical supervision. Residential: Training of kitchen staff is required.
Dairy-based products and eggs	Whole milk, Skim milk powder, cheese, Greek yogurt, whole eggs, egg white powder	High biological value protein, familiar to most elderly, and easily incorporated into food.	High *(Mentioned in the ESPEN 2022 guidelines [30] and Geny et al., 2023 systematic review of literature [52])*	Community: Relies on caregiver or patient to purchase, measure, and mix ingredients accurately. Residential: Compliance requires routine monitoring of individual intake.
Plant-based products	Avocados, pureed/powdered nuts, nut butters, pulses/legumes, semolina	Provides dietary diversity and healthy fats and potential additional health benefits (e.g., phytonutrient compounds).	Moderate *(Mentioned in the Geny et al., 2023 systematic review of literature [52])*	Community: Requires preparation skills and some items (e.g., avocados) may have shorter shelf-lives. Residential: Lower acceptability of unfamiliar food items, may requires recipe adaptations.
Macronutrient extracts	Whey protein isolate, milk protein concentrate, caseinates, maltodextrin	Highly concentrated, can be added to liquids without significantly changing volume or taste.	High *(Mentioned in the ESPEN 2022 guidelines [30] and Geny et al., 2023 systematic review of literature [52])*	Community: Can be expensive and not readily available. Education on usage is required. Residential: Compliance requires monitoring individual intake.

### 3.2. Oral Nutritional Supplements: Clinical Efficacy and Economic Impact

When fortified food alone fails to meet nutritional targets, ONSs become an essential component of the care pathway. According to the ESPEN 2022 guidelines, older individuals experiencing malnutrition or at nutritional risk with underlying chronic conditions should receive ONSs when dietary counselling and food enrichment strategies are inadequate to improve intake and achieve nutritional goals [30]. ONSs are formulated to deliver concentrated sources of energy, protein and nutrients in multiple forms, such as ready-to-consume liquids, crèmes, or powdered supplements that can be prepared as drinks or mixed into food and drink [57], allowing them to serve as an effective strategy for meeting protein quantity and quality recommendations while helping to maintain an evenly distributed protein intake pattern throughout the day.

An RCT conducted in Seoul, South Korea [58] enrolled 87 frail older adults with low socioeconomic status to investigate the preventive impact of protein-energy supplementation on functional decline. The intervention group maintained stable physical performance and gait speed while controls experienced significant deterioration. Specifically, supplementation preserved Short Physical Performance Battery scores (*p* = 0.039), usual gait speed (*p* = 0.039), and improved timed up-and-go performance (*p* = 0.038) compared to controls, with a trend toward better physical functioning (*p* = 0.052). Hand grip strength and balance showed no between-group differences [58]. However, a systematic review and meta-analysis of 12 RCTs which also included the Kim and Lee (2013) study assessed ONS effectiveness among community-dwelling older adults reported that supplementation resulted in significant increases in energy intake, BW, BMI, and calf circumference as well as functional outcomes also improved significantly, with notable gains in grip strength and minor changes in walking speed [59]. Beyond improvements in dietary intake and functional outcomes, ONSs have demonstrated additional clinical and economic benefits. A 2020 UK-based randomised controlled trial included 308 malnourished older adults assigned to receive either ONSs combined with dietary advice (DA) or DA alone. The study evaluated effects on intake, BW, QoL, healthcare utilisation, and participant satisfaction. The ONS plus DA group achieved significantly greater total energy and protein intakes and greater weight gain compared to DA alone. QoL improved over time in both groups. However, the ONS plus DA group demonstrated significantly greater improvements in QoL index. Notably, ONSs plus DA substantially reduced healthcare utilisation, with reductions of 34% in healthcare practitioner visits, 50% in emergency admissions, and 62% in length of hospital stay. While both interventions demonstrated high acceptability (ONSs 96%, DA 95%), participant satisfaction was significantly higher with ONSs (89%) than DA (73%) [60].

Despite evidence that ONSs can enhance energy and protein intake and functional outcomes, considerable methodological heterogeneity across studies can limit the extent to which their independent clinical benefits can be established. A key issue is the difference in intervention duration, which ranges from approximately 2 to 12 months, raising uncertainty over whether early improvements are maintained and reflected in favourable changes in body composition (e.g., increased FFM) and functional status. Interpretation is further complicated by inconsistent approaches to measuring adherence. Studies have used different compliance thresholds (e.g., 70% or 80%) of prescribed intake, while relying on self-reported consumption, returned product counts, or staff records. Such differences may introduce bias and limit comparability across studies. Moreover, variation in the initial nutritional status of participants (e.g., variability in nutritional screening tools) makes it difficult to determine whether ONSs are equally effective across different severity of malnutrition or frailty, particularly where outcomes are not reported according to baseline nutritional status. Finally, many ONS interventions are delivered alongside other interventions such as dietary advice and specific nutritional supplementation (e.g., vitamin D and HMB), making it difficult to determine the independent contributions of ONSs. Therefore, future research must address these gaps through adequately powered, well-designed trials specifically targeting care home populations, while incorporating robust functional assessments. Furthermore, a 2016 systematic review examining 19 publications on the cost effectiveness of ONSs, primarily based on RCTs, indicated that the use of standard ONSs in community settings, whether alone or combined with hospital use, results in overall net cost savings favouring the ONS group or achieves cost neutrality [61]. Meta-analysis showed that ONSs significantly decreased hospitalisation by 16.5% with a non-significant trend toward reduced mortality [61]. Additionally, these economic outcomes were accompanied by clinically significant benefits including enhanced QoL, reduced infection rates, fewer minor post-operative complications, decreased fall incidence, and improved functional capacity [61]. Also, consistent cost advantages of the ONS groups were seen, regardless of stratification by nutritional risk, age, study design, intervention length, or care environment [61]. From a cost-effectiveness perspective, such economic analyses are compelling; however, its impact in the real-world settings may vary according to available resources and consistency. Implementation may lead to additional costs related to staff time, training, and product management (e.g., storage and administration). Such approaches may be difficult to sustain where staffing and resources are limited, therefore, projected cost saving observed in controlled research settings may not be readily replicated in routine care, highlighting the need for care home-specific economic evaluations. Another emerging consideration is the use of plant-based ONSs as an alternative to conventional formulations. A recent multicentre prospective study based in the UK found that plant-based ONSs may provide a viable nutritional support option for individuals at risk of malnutrition, with significant improvements in nutritional status reported following supplementations [62]. Similarly, a 2025 RCT conducted in Spain reported that plant-based ONSs were compatible to conventional formulations in terms of nutritional outcomes amongst malnourished patients [63]. However, evidence base remains limited, and the average age of the participants in both studies was 59–60 years, therefore further comparative research particularly with older care home populations is warranted to determine optimal formulations, acceptability, and long-term effectiveness.

### 3.3. Implementation Science: Adherence, Tolerability, and Support

ONSs provide a clinically proven and relatively affordable approach to dietary supplementation for individuals with or at risk of malnutrition. However, the gap between nutritional prescription and actual consumption represents a common point of intervention failure. While adherence (or compliance) is essential for achieving clinical effectiveness, it poses significant challenges, particularly among vulnerable populations such as undernourished older adults [64,65]. Hence, understanding the various factors that influence ONS intake is crucial for enhancing the effectiveness of nutritional interventions [64,65].

A 2012 systematic review of 46 studies reported mean compliance rates of 78% across all ONS regimens and settings (ranging from 37% to 100%), with hospitalised patients showing lower compliance (67%) than community-dwelling elderly individuals (81%). Notably, compliance was particularly favourable for higher energy density ONS formulations [66]. Findings from this systematic review also indicated that larger ONS prescriptions (in terms of energy) were significantly associated with greater intake though not with overall compliance rates. This may reflect clinical judgment of the prescribing healthcare professional [66]. Therefore, prescriptions should be regularly monitored and adjusted accordingly, either reduced to minimise waste or increased to meet nutritional requirements [66]. Given the potential influence on appetite, the timing of ONS administration relative to meals may impact both adherence and total nutritional intake. ESPEN advises that ONSs should be provided between meals [30]. Also, research by Hubbard et al. demonstrated satisfactory compliance with between-meal administration by staff, yet it was found that permitting “ad libitum” consumption yielded higher adherence rates [66]. Furthermore, a 2022 systematic review of 10 studies investigating the Medication Pass Nutritional Supplement Program (MEDPass) (which administers ONSs in unusually small, frequent doses (three to four times daily) during medication rounds) found that compliance improved by 23.4% to 66%, achieving overall adherence rates from 72.7% to 96% in hospitalised adults and nursing home residents [67]. Likewise, several external factors have been identified as influencing ONS adherence. Social isolation in older adults, stemming from reduced social networks and limited mobility, contributes to undernutrition risk [64]. Evidence suggests that communal dining environments improves nutritional intake, BW, and ONS adherence compared to eating alone, highlighting the importance of social eating contexts in this population [64].

Moreover, healthcare professionals, family members, and carers play critical roles in supporting ONS adherence. Since family members often undervalue ONS compared to other nutritional interventions, education regarding its benefits is important [64]. A 2025 systematic mixed-studies review of 171 articles revealed that several studies identified healthcare professionals’ insufficient knowledge and confidence regarding ONSs created barriers to patient consumption, as a result of gaps in fundamental nutritional understanding, ONS-specific information, and training [65]. However, the study also indicates that improved nutritional awareness among healthcare staff, achieved through education on ONS’s role and establishing appropriate nutritional care perspectives, could facilitate better adherence. Additionally, adopting a person-centred approach that individualises and tailors ONS prescriptions according to patient needs and ensures appropriate monitoring and follow-up may further enhance compliance [65]. Figure 2 below depicts the multiple interacting factors affecting ONS adherence. 

**Figure 2:**
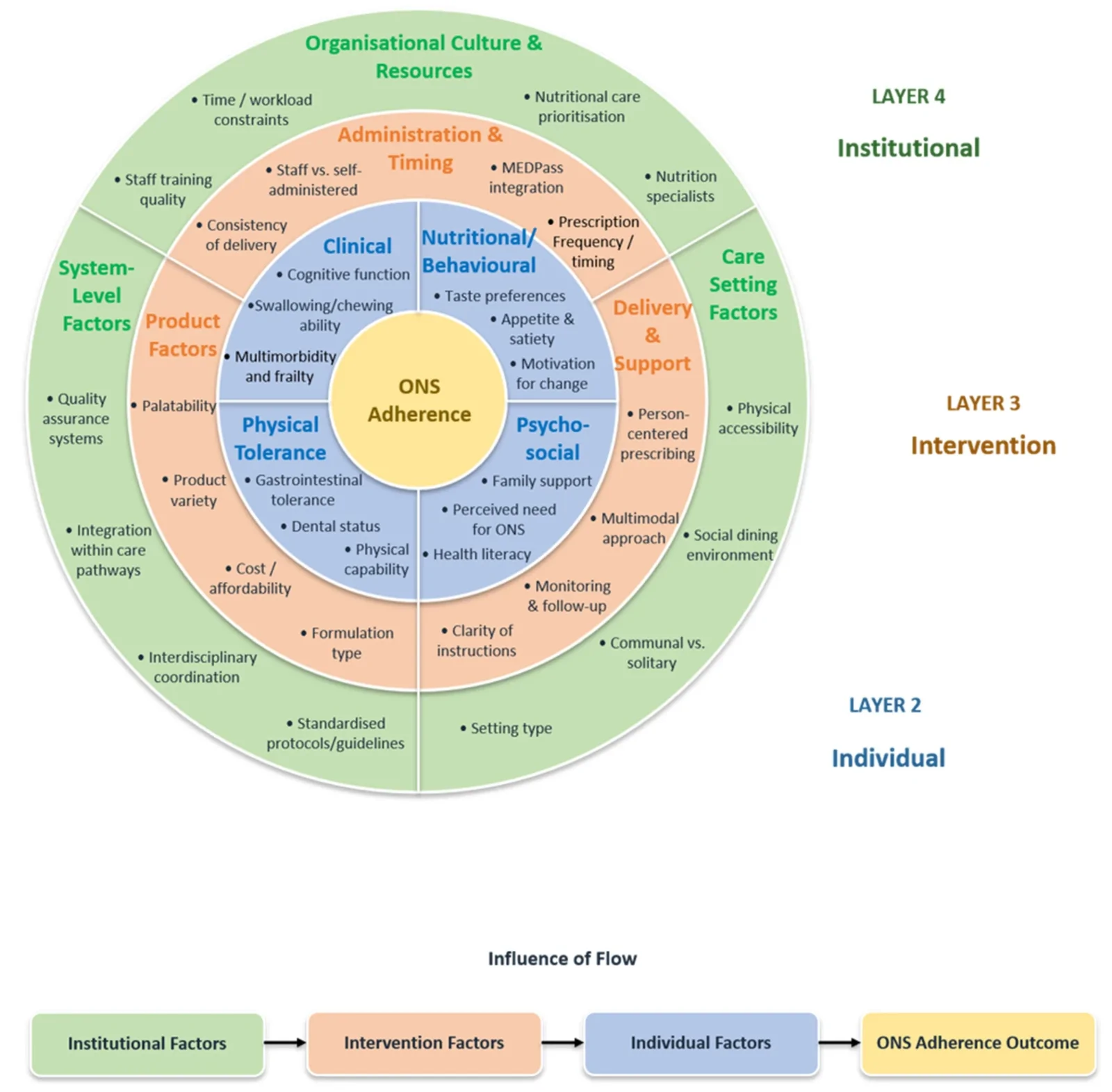
Concentric framework of factors influencing oral nutritional supplement (ONS) adherence. This concentric model conceptualises patient ONS adherence not based upon isolated behavioural factors, but as an outcome of dynamic, layered interactions. At the core, adherence and compliance represent the primary clinical outcome, with an aim to improve nutritional status. Surrounding the core, intrinsic individual parameters including frailty severity and multimorbidity, appetite, taste preferences, and health literacy are key physical and psychological factors. These intrinsic factors are continuously modified by intervention-level characteristics, where product palatability and structured administration protocols (e.g., MEDPass timing) directly buffer patient-level barriers. Crucially, these clinical and intervention processes are bounded by macro-level institutional drivers, including organisational culture, staffing capacity, care setting environment, and standardised screening pathways (e.g., malnutrition screening such as MNA-SF, GLIM). Downstream compliance failures thus could also be frequently rooted in systemic organisational constraints rather than just primary individual non-compliance. Hence, enhanced adherence to ONS must be contextualised within comprehensive screening and assessment, multimodal intervention, and person-centred goals prioritising functional outcomes and quality of life over improving body weight alone.

### 3.4. Nutrition as a Key Component of a Multi-Modal Intervention in Frail Care Home Residents

As outlined, nutrition interventions have the potential to improve key outcomes, and this may also be possible in those who are already frail and pre-frail. A recent systematic review and meta-analysis of nutritional management interventions (13 studies analysed) showed that interventions can significantly improve dietary protein intake, BW, and gait speed in those who are either frail or pre-frail [68]. An updated 2026 systematic review analysing 11 randomised control trials on the effects of nutritional guidance on frailty found that long-term nutritional guidance programs, especially when integrated with physical activity, yielded more consistent frailty improvements. Furthermore, it was noted that individually tailored interventions delivered by trained professionals proved most effective [69].

There are many complex factors that may impact upon intervention success and improvement in outcomes, and hence there is a strong rationale for using a multimodal/component intervention strategy. This may include the addition of physical activity and exercise for stimulation of neuromuscular activity and muscle anabolic responses and psychosocial interventions to improve adherence and motivation, as well as potential drug treatment (e.g., medications to improve appetite and/or anabolic responses). One major issue however is the lack of current available evidence and research from care home and residential care settings, as outlined in this recent scoping review [70]. In the field of wasting cachexia in chronic disease (e.g., cancer, COPD, chronic kidney disease), evidence from a recent Cochrane systematic review concluded that multidisciplinary approaches (e.g., nutrition, exercise, with anti-inflammatory, anabolic and appetite stimulatory drug treatments) may be more effective for improving clinical outcomes, although further high-quality research is needed to confirm their effectiveness [71]. This is important research to consider as many care home residents may be living with multimorbidity and chronic disease conditions impacting their nutritional status, QoL, and physical function. Future care home studies will be necessary to determine the impact of multimodal interventions incorporating aspects such as ONSs, physical activity, and other key components (e.g., medications/drugs, and psychosocial/behavioural).

## 4. Conclusions

The global demographic shift toward an ageing population presents significant public health challenges, with increased prevalence of chronic diseases and functional decline. Malnutrition, sarcopenia, and frailty frequently coexist in this vulnerable population, particularly among care home residents, where prevalence rates are substantially high.

Nutritional strategies represent a cornerstone of preventive and therapeutic care for older adults at risk of or experiencing malnutrition. The Food First approach forms the fundamental basis of adult nutrition care, while also sustaining identity, pleasure, and cultural aspects. ONSs serve as an essential component of the nutritional care pathway when dietary counselling and food fortification prove insufficient, with demonstrated benefits including improved BW, functional capacity, QoL, reduced infection rates, as well as fewer hospital admissions making ONSs a cost-effective solution.

However, the success of these interventions heavily relies on addressing these complex, interrelated conditions with a holistic individualised approach. Optimal outcomes may be achieved when nutritional interventions are integrated into a comprehensive multimodal approach alongside appropriate exercise programmes, medication review and pharmacological interventions, and psychosocial and behavioural strategies (e.g., to improve adherence). Furthermore, it may also be critical to implement appropriate and accurate screening and assessment strategies to correctly ascertain intervention effects and success, e.g., measurement of appropriate outcomes such as muscle mass, strength and function, along with QoL indicators, together with early screening and improving adherence through staff and family education, regular monitoring, and focusing on providing a personalised, patient-centred care approach for older adults.

Despite growing evidence, significant gaps remain in understanding the ideal implementation of combined nutritional strategies in care home settings. This review highlights the rationale for further research to evaluate the comparative effectiveness of different nutritional approaches and the importance of establishing standardised protocols that can be consistently applied across diverse care environments to optimise health outcomes and QoL for older adults.

## Data Availability

No new data were created or analysed in this study. Data sharing is not applicable to this article.

## References

[1] World Health Organization Ageing and Health 2025. https://www.who.int/news-room/fact-sheets/detail/ageing-and-health.

[2] Iburg K.M., Charalampous P., Allebeck P., Stenberg E.J., O’caoimh R., Monasta L., Peñalvo J.L., Pereira D.M., A Wyper G.M., Niranjan V. (2023). Burden of disease among older adults in Europe—Trends in mortality and disability, 1990–2019. Eur. J. Public Health.

[3] Valabhji J., Barron E., Pratt A., Hafezparast N., Dunbar-Rees R., Turner E.B., Roberts K., Mathews J., Deegan R., Cornelius V. (2024). Prevalence of multiple long-term conditions (multimorbidity) in England: A whole population study of over 60 million people. J. R. Soc. Med..

[4] Norman K., Haß U., Pirlich M. (2021). Malnutrition in older adults—Recent advances and remaining challenges. Nutrients.

[5] Tu Y., Garden G., Wilkinson L., Slee A. (2023). The prevalence of malnutrition (MUST and MNA-SF), frailty and physical disability and relationship with mortality in older care home residents. Clin. Nutr. Open Sci..

[6] Office for National Statistics (2023). Older People Living in Care Homes in 2021 and Changes Since 2011. https://www.ons.gov.uk/peoplepopulationandcommunity/birthsdeathsandmarriages/ageing/articles/olderpeoplelivingincarehomesin2021andchangessince2011/2023-10-09.

[7] Department of Health and Social Care (2025). Adult Social Care in England, Monthly Statistics: December 2025. https://www.gov.uk/government/statistics/adult-social-care-in-england-monthly-statistics-december-2025/adult-social-care-in-england-monthly-statistics-december-2025.

[8] Hegazi R., Miller A., Sauer A. (2024). Evolution of the diagnosis of malnutrition in adults: A primer for clinicians. Front. Nutr..

[9] Cederholm T., Jensen G., Correia M., Gonzalez M., Fukushima R., Higashiguchi T., Baptista G., Barazzoni R., Blaauw R., Coats A. (2019). GLIM criteria for the diagnosis of malnutrition–a consensus report from the global clinical nutrition community. J. Cachexia Sarcopenia Muscle.

[10] Roberts H.C., Lim S.E., Cox N.J., Ibrahim K. (2019). The challenge of managing undernutrition in older people with frailty. Nutrients.

[11] Cox N.J., Morrison L., Ibrahim K., Robinson S.M., Sayer A.A., Roberts H.C. (2020). New horizons in appetite and the anorexia of ageing. Age Ageing.

[12] Age UK (2023). Tackling Malnutrition. https://www.ageuk.org.uk/discover/2023/november/tackling-malnutrition/.

[13] British Association for Parenteral and Enteral Nutrition (2022). Malnutrition and Nutritional Care Survey in Adults.

[14] Cruz-Jentoft A.J., Baeyens J.P., Bauer J.M., Boirie Y., Cederholm T., Landi F., Martin F.C., Michel J.P., Rolland Y., Schneider S.M. (2010). Sarcopenia: European consensus on definition and diagnosis: Report of the European Working Group on Sarcopenia in Older People. Age Ageing.

[15] Cruz-Jentoft A.J., Bahat G., Bauer J., Boirie Y., Bruyère O., Cederholm T., Cooper C., Landi F., Rolland Y., Sayer A.A. (2019). Sarcopenia: Revised European consensus on definition and diagnosis. Age Ageing.

[16] Faulkner J.A., Larkin L.M., Claflin D.R., Brooks S.V. (2007). Age-related changes in the structure and function of skeletal muscles. Clin. Exp. Pharmacol. Physiol..

[17] Manini T.M., Clark B.C. (2012). Dynapenia and aging: An update. J. Gerontol. Ser. A Biomed. Sci. Med. Sci..

[18] Wei W.-X., Mao Z.-F., Chen M.-L., Meng L. (2025). The impact of chronic diseases and lifestyle on sarcopenia risk in older adults: A population-based longitudinal study. Front. Med..

[19] Papadopoulou S.K. (2020). Sarcopenia: A contemporary health problem among older adult populations. Nutrients.

[20] Papadopoulou S., Tsintavis P., Potsaki G., Papandreou D. (2020). Differences in the prevalence of sarcopenia in community-dwelling, nursing home and hospitalized individuals. A systematic review and meta-analysis. J. Nutr. Health Aging.

[21] Shen Y., Chen J., Chen X., Hou L., Lin X., Yang M. (2019). Prevalence and associated factors of sarcopenia in nursing home residents: A systematic review and meta-analysis. J. Am. Med. Dir. Assoc..

[22] Shafiee G., Keshtkar A., Soltani A., Ahadi Z., Larijani B., Heshmat R. (2017). Prevalence of sarcopenia in the world: A systematic review and meta-analysis of general population studies. J. Diabetes Metab. Disord..

[23] Fried L.P., Tangen C.M., Walston J., Newman A.B., Hirsch C., Gottdiener J., Seeman T., Tracy R., Kop W.J., Burke G. (2001). Frailty in older adults: Evidence for a phenotype. J. Gerontol. Ser. A Biol. Sci. Med. Sci..

[24] Kim D.H., Rockwood K. (2024). Frailty in older adults. N. Engl. J. Med..

[25] Galluzzo L., O’Caoimh R., Rodríguez-Laso Á., Beltzer N., Ranhoff A.H., Van der Heyden J., Lamprini-Koula M., Ciutan M., Lopez-Samaniego L., Liew A. (2018). Incidence of frailty: A systematic review of scientific literature from a public health perspective. Ann. Dell’istituto Super. Sanita.

[26] Cruz-Jentoft A.J., Kiesswetter E., Drey M., Sieber C.C. (2017). Nutrition, frailty, and sarcopenia. Aging Clin. Exp. Res..

[27] Taylor J.A., Greenhaff P.L., Bartlett D.B., Jackson T.A., Duggal N.A., Lord J.M. (2023). Multisystem physiological perspective of human frailty and its modulation by physical activity. Physiol. Rev..

[28] Martone A.M., Bernabei R. (2015). Sarcopenia as the biological substrate of physical frailty. Clin. Geriatr. Med..

[29] Verlaan S., Ligthart-Melis G.C., Wijers S.L., Cederholm T., Maier A.B., de van der Schueren M.A. (2017). High prevalence of physical frailty among community-dwelling malnourished older adults–a systematic review and meta-analysis. J. Am. Med. Dir. Assoc..

[30] Volkert D., Beck A.M., Cederholm T., Cruz-Jentoft A., Hooper L., Kiesswetter E., Maggio M., Raynaud-Simon A., Sieber C., Sobotka L. (2022). ESPEN practical guideline: Clinical nutrition and hydration in geriatrics. Clin. Nutr..

[31] Cederholm T., Jensen G.L., Correia M.I.T., Gonzalez M.C., Fukushima R., Pisprasert V., Blaauw R., Braz D.C., Carrasco F., Jentoft A.J.C. (2025). The GLIM consensus approach to diagnosis of malnutrition: A 5-year update. Clin. Nutr..

[32] Power L., de van der Schueren M.A., Leij-Halfwerk S., Bauer J., Clarke M., Visser M., Volkert D., Bardon L., Gibney E., Corish C.A. (2019). Development and application of a scoring system to rate malnutrition screening tools used in older adults in community and healthcare settings–A MaNuEL study. Clin. Nutr..

[33] Leij-Halfwerk S., Verwijs M.H., van Houdt S., Borkent J.W., Guaitoli P., Pelgrim T., Heymans M.W., Power L., Visser M., Corish C.A. (2019). Prevalence of protein-energy malnutrition risk in European older adults in community, residential and hospital settings, according to 22 malnutrition screening tools validated for use in adults ≥ 65 years: A systematic review and meta-analysis. Maturitas.

[34] NICE Nutrition Support for Adults: Oral Nutrition Support, Enteral Tube Feeding and Parenteral Nutrition 2006. https://www.nice.org.uk/guidance/cg32/chapter/Recommendations.

[35] Mohammad Z., Evans S., Kassem H. (2025). Nutritional screening in older adults: A MUST. Clin. Nutr. ESPEN.

[36] Volkert D., Beck A.M., Cederholm T., Cereda E., Cruz-Jentoft A., Goisser S., de Groot L., Großhauser F., Kiesswetter E., Norman K. (2019). Management of malnutrition in older patients—Current approaches, evidence and open questions. J. Clin. Med..

[37] Rubenstein L.Z., Harker J.O., Salvà A., Guigoz Y., Vellas B. (2001). Screening for undernutrition in geriatric practice: Developing the short-form mini-nutritional assessment (MNA-SF). J. Gerontol. Ser. A Biol. Sci. Med. Sci..

[38] Kaiser M.J., Bauer J.M., Ramsch C., Uter W., Guigoz Y., Cederholm T., Thomas D.R., Anthony P., Charlton K.E., Maggio M. (2009). Validation of the Mini Nutritional Assessment short-form (MNA^®^-SF): A practical tool for identification of nutritional status. J. Nutr. Health Aging.

[39] Valentini A., Federici M., Cianfarani M.A., Tarantino U., Bertoli A. (2018). Frailty and nutritional status in older people: The Mini Nutritional Assessment as a screening tool for the identification of frail subjects. Clin. Interv. Aging.

[40] Soysal P., Isik A.T., Arik F., Kalan U., Eyvaz A., Veronese N. (2019). Validity of the mini-nutritional assessment scale for evaluating frailty status in older adults. J. Am. Med. Dir. Assoc..

[41] Li L., Xia Z., Zeng X., Tang A., Wang L., Su Y. (2024). The agreement of different techniques for muscle measurement in diagnosing sarcopenia: A systematic review and meta-analysis. Quant. Imaging Med. Surg..

[42] Bauer J., Biolo G., Cederholm T., Cesari M., Cruz-Jentoft A.J., Morley J.E., Phillips S., Sieber C., Stehle P., Teta D. (2013). Evidence-based recommendations for optimal dietary protein intake in older people: A position paper from the PROT-AGE Study Group. J. Am. Med. Dir. Assoc..

[43] Coelho-Júnior H.J., Rodrigues B., Uchida M., Marzetti E. (2018). Low protein intake is associated with frailty in older adults: A systematic review and meta-analysis of observational studies. Nutrients.

[44] Borkent J., Manders M., Nijhof A., Wijker L., Feskens E., Naumann E., de Van Der Schueren M. (2023). Too low protein and energy intake in nursing home residents. Nutrition.

[45] Blanquet M., Guiguet-Auclair C., Berland P., Ducher G., Sauvage A., Dadet S., Guiyedi V., Farigon N., Bohatier J., Gerbaud L. (2023). Are energy and protein intakes lower than requirements in older adults? An urgent issue in hospitals and nursing homes. Nutrients.

[46] Kim I.-Y., Schutzler S., Schrader A.M., Spencer H.J., Azhar G., Wolfe R.R., Ferrando A.A. (2018). Protein intake distribution pattern does not affect anabolic response, lean body mass, muscle strength or function over 8 weeks in older adults: A randomized-controlled trial. Clin. Nutr..

[47] Loenneke J.P., Loprinzi P.D., Murphy C.H., Phillips S.M. (2016). Per meal dose and frequency of protein consumption is associated with lean mass and muscle performance. Clin. Nutr..

[48] Ely I.A., Phillips B.E., Smith K., Wilkinson D.J., Piasecki M., Breen L., Larsen M.S., Atherton P.J. (2023). A focus on leucine in the nutritional regulation of human skeletal muscle metabolism in ageing, exercise and unloading states. Clin. Nutr..

[49] Marshall R.N., Smeuninx B., Morgan P.T., Breen L. (2020). Nutritional strategies to offset disuse-induced skeletal muscle atrophy and anabolic resistance in older adults: From whole-foods to isolated ingredients. Nutrients.

[50] Harris S., DePalma J., Barkoukis H. (2025). Protein and aging: Practicalities and practice. Nutrients.

[51] The Malnutrition Pathway (2021). Managing Adult Malnutrition in the Community.

[52] Geny A., Petitjean M., Van Wymelbeke-Delannoy V., Sulmont-Rossé C. (2023). Impact of food-based fortification on nutritional outcomes and acceptability in older adults: Systematic literature review. Front. Nutr..

[53] Beelen J., de Roos N.M., De Groot L. (2017). Protein enrichment of familiar foods as an innovative strategy to increase protein intake in institutionalized elderly. J. Nutr. Health Aging.

[54] Douglas J.W., Lawrence J.C., Knowlden A.P. (2017). The use of fortified foods to treat malnutrition among older adults: A systematic review. Qual. Ageing Older Adults.

[55] Pölönen S., Tiihonen M., Hartikainen S., Nykänen I. (2017). Individually tailored dietary counseling among old home care clients-effects on nutritional status. J. Nutr. Health Aging.

[56] Morilla-Herrera J.C., Martín-Santos F.J., Caro-Bautista J., Saucedo-Figueredo C., García-Mayor S., Morales-Asencio J.M. (2016). Effectiveness of food-based fortification in older people a systematic review and meta-analysis. J. Nutr. Health Aging.

[57] Cederholm T., Barazzoni R., Austin P., Ballmer P., Biolo G., Bischoff S.C., Compher C., Correia I., Higashiguchi T., Holst M. (2017). ESPEN guidelines on definitions and terminology of clinical nutrition. Clin. Nutr..

[58] Kim C.-O., Lee K.-R. (2013). Preventive effect of protein-energy supplementation on the functional decline of frail older adults with low socioeconomic status: A community-based randomized controlled study. J. Gerontol. Ser. A Biomed. Sci. Med. Sci..

[59] Asghari Hanjani N., Arzhang P., Azadbakht L. (2025). Effect of oral nutrition supplements on anthropometric and functional parameters among community-dwelling older adults: A systematic review and meta-analysis of randomized controlled trials. BMC Nutr..

[60] Smith T.R., Cawood A.L., Walters E.R., Guildford N., Stratton R.J. (2020). Ready-made oral nutritional supplements improve nutritional outcomes and reduce health care use—A randomised trial in older malnourished people in primary care. Nutrients.

[61] Elia M., Normand C., Laviano A., Norman K. (2016). A systematic review of the cost and cost effectiveness of using standard oral nutritional supplements in community and care home settings. Clin. Nutr..

[62] Delsoglio M., Griffen C., Syed R., Cookson T., Saliba H., Vowles A., Davies S., Willey N., Thomas J., Millen N. (2023). A multi-center prospective study of plant-based nutritional support in adult community-based patients at risk of disease-related malnutrition. Front Nutr..

[63] Iglesias Hernández N.C., Ramos Carrasco A., De Luis Román D.A., de Pablos-Velasco P.L., Calañas Continente A., Rodríguez Soto M.J., López-Gómez J.J., Comi-Díaz C., García-Rey S., Pérez-Rambla C. (2025). Effect of a new plant-based high-energy oral nutritional supplement in adult malnourished patients: An open-label, randomized clinical trial. Front Nutr..

[64] Lester S., Kleijn M., Cornacchia L., Hewson L., Taylor M., Fisk I. (2022). Factors affecting adherence, intake, and perceived palatability of oral nutritional supplements: A literature review. J. Nutr. Health Aging.

[65] Liljeberg E., Payne L., Josefsson M.S., Söderström L., Einarsson S. (2025). Understanding the complexity of barriers and facilitators to adherence to oral nutritional supplements among patients with malnutrition: A systematic mixed-studies review. Nutr. Res. Rev..

[66] Hubbard G.P., Elia M., Holdoway A., Stratton R.J. (2012). A systematic review of compliance to oral nutritional supplements. Clin. Nutr..

[67] Krebs F., Uhlmann K., Stanga Z., Kurmann S. (2022). Distribution of oral nutritional supplements with medication: Is there a benefit? A systematic review. Nutrition.

[68] Li W., Wu Z., Liao X., Geng D., Yang J., Dai M., Talipti M. (2024). Nutritional management interventions and multi-dimensional outcomes in frail and pre-frail older adults: A systematic review and meta-analysis. Arch. Gerontol. Geriatr..

[69] Koyama T., Nohara J., Nakamura M. (2026). Effects of nutritional guidance on frailty in older adults: A systematic review. J. Nutr. Health Aging.

[70] Ambagtsheer R., Leach M., O’Brien L., Tyndall J., Wardle J., Beilby J. (2024). Multidisciplinary, multicomponent interventions to reduce frailty among older persons in residents of residential care facilities: A scoping review. Syst. Rev..

[71] Reid J., Blair C., Dempster M., McKeaveney C., Slee A., Fitzsimons D. (2025). Multimodal interventions for cachexia management. Cochrane Database Syst. Rev..

